# Grain size effect on the radiation damage tolerance of cubic zirconia against *simultaneous* low and high energy heavy ions: Nano triumphs bulk

**DOI:** 10.1038/s41598-021-90214-6

**Published:** 2021-05-25

**Authors:** Parswajit Kalita, Santanu Ghosh, Gaëlle Gutierrez, Parasmani Rajput, Vinita Grover, Gaël Sattonnay, Devesh K. Avasthi

**Affiliations:** 1 Department of Physics, Indian Institute of Technology Delhi, New Delhi, 110016 India; 2 School of Engineering, University of Petroleum and Energy Studies, Dehradun, 248007 India; 3 CEA Saclay, DEN, SRMP, Labo JANNUS, 91191 Gif-sur-Yvette, France; 4 Beamline Development and Application Section, Bhabha Atomic Research Centre, Mumbai, 400085 India; 5 Chemistry Division, Bhabha Atomic Research Centre, Mumbai, 400085 India; 6 Université Paris Saclay, CNRS/IN2P3, IJC Lab, 91405 Orsay, France

**Keywords:** Materials science, Nanoscience and technology, Physics

## Abstract

Irradiation induced damage in materials is highly detrimental and is a critical issue in several vital science and technology fields, e.g., the nuclear and space industries. While the effect of dimensionality (nano/bulk) of materials on its radiation damage tolerance has been receiving tremendous interest, studies have only concentrated on low energy (nuclear energy loss (S_n_) dominant) and high energy (electronic energy loss (S_e_) dominant) irradiations independently (wherein, interestingly, the effect is opposite). In-fact, research on radiation damage in general has almost entirely focused only on independent irradiations with low and/or high energy particles till date, and investigations under simultaneous impingement of energetic particles (which also correspond to the actual irradiation conditions during real-world applications) are very scarce. The present work elucidates, taking cubic zirconia as a model system, the effect of grain size (26 nm vs 80 nm) on the radiation tolerance against *simultaneous* irradiation with low energy (900 keV I) and high energy (27 meV Fe) particles/ions; and, in particular, introduces the enhancement in the radiation damage tolerance upon downsizing from bulk to nano dimension. This result is interpreted within the framework of the thermal-spike model after considering (1) the fact that there is essentially no spatial and time overlap between the damage events of the two ‘simultaneous’ irradiations, and (2) the influence of grain size on radiation damage against individual S_n_ and S_e_. The present work besides providing the first fundamental insights into how the grain size/grain boundary density inherently mediates the radiation response of a material to simultaneous S_n_ and S_e_ deposition, also (1) paves the way for potential application of nano-crystalline materials in the nuclear industry (where simultaneous irradiations with low and high energy particles correspond to the actual irradiation conditions), and (2) lays the groundwork for understanding the material behaviour under other simultaneous (viz. S_n_ and S_n_, S_e_ and S_e_) irradiations.

## Introduction

Exposure of materials to energetic particles/ions, i.e. irradiation, often results in the creation of defects and subsequent micro-structural changes in the material, eventually leading to a degradation of its properties (i.e. radiation damage). Such exposure of materials to energetic particles is thus a detrimental process. This is a critical issue in several vital applications, e.g. nuclear energy (fission & fusion), electronic devices in space vehicles, particle detectors, semiconductor doping etc.^1–7^, wherein materials are subjected to severe irradiation with low energy and/or high energy particles (predominantly slowing down by ballistic/nuclear collisions (S_n_)^8^ and electronic excitations (S_e_)^8^ respectively) during service. For these applications, understanding the behaviour of the materials under irradiation, and thus designing them to be more radiation damage tolerant, is therefore crucial. A common way to simulate the effects of such irradiations, within a limited time, is to use energetic ion beams from accelerators^2,9^. Note, however, that for such studies aimed at understanding the radiation response to be truly relevant, it is crucial that they are carried out at the corresponding in-service (irradiation) conditions.

In the recent past, nano-scale materials design, i.e. downsizing of materials to nano-dimension, has received significant attention and is being considered as an effective strategy in the context of mitigating the radiation damage in materials. It has been observed that the nano-crystalline (NC) state exhibits enhanced radiation damage tolerance, against low energy ions (S_n_ dominant), when compared to its bulk counterparts^5,10–20^. We have, however, very recently shown that the better radiation tolerance of NC materials is not always true and designing nano-scale materials in order to lower the irradiation induced damage is not advantageous against high energy ions (S_e_ dominant)^21^. In other words, our results^21^ show that the situation is very different under high energy irradiations and the NC phase is more damaged as compared to its bulk(-like) counterpart. A similar behaviour has been observed in the case of Ceria irradiated with high energy ions as well^22^. Note here that the damage creation mechanisms under low energy and high energy irradiations are fundamentally very different - in the low energy regime, the incident ions primarily undergo elastic collisions with the target atoms (referred to as nuclear energy loss (S_n_)) and subsequently produce lattice disorder/damage by the displacement of the atoms from their respective positions in collision cascades^8^; on the other hand, in the high energy regime, the interaction of the incident ions with the target atoms is predominantly inelastic (referred to as electronic energy loss (S_e_)) and the subsequent damage production is via/due to the transient rise and fall in the lattice temperature (‘thermal spike’ mechanism)^8^. This contrary dependence of the radiation damage tolerance on the grain size of the material, based on the energy loss mechanism (i.e., S_n_ or S_e_) of the incident particles, thus raises an intriguing fundamental question, viz. what will be the effect of grain size on the radiation tolerance against simultaneous S_n_ and S_e_? Will the effect be similar to that against low energy irradiations and the NC state be more radiation tolerant; or will the effect be more inclined towards the high energy irradiation results and the NC phase be more damaged? In either case, why? Or will the effect be something totally different?

It should be emphasized here that understanding the grain size mediated radiation response under concomitant S_n_ and S_e_, apart from being of fundamental interest, is also mandatory from an application point of view since materials used in nuclear reactors (e.g. fuels, IMFs^23^) and/or waste matrices are actually exposed to simultaneous (and *not* independent) irradiation with low energy (alpha recoils) and high energy (fission fragments) particles. Here, the importance of nuclear energy in fulfilling our energy requirements, particularly in the light of rapidly depleting fossil fuel reserves and climate change, is a point worth considering.

Despite the decades of research devoted to understanding the radiation damage in materials, studies concentrating on the effects of simultaneous irradiations are very scarce^1,2,9,24^; i.e. the effects of independent/individual and sequential irradiation with low and high energy ions have been studied extensively and are well understood (see e.g. Refs.^1,8,25^ and references therein), but investigations upon simultaneous impingement of energetic particles (which are of actual relevance from the perspective of real-world applications^2^) are very limited. In a recent pioneering work, Thome et al.^1^ had reported the effects of simultaneous S_n_ and S_e_ irradiation on various classes of materials. More recently, simultaneous irradiation studies on silica and UO_2_ have been reported^2,9^. The materials in the studies^1,24^ of Thome and co-workers were all in the *single-crystalline* state, and moreover the studies were, to our best understanding, aimed at examining (possible) combined/co-operative effects of S_n_ and S_e_. On the other hand, the silica, in the report by Mir et al.^2^, was in the *amorphous* form, and furthermore the aim of this particular study was, in our best interpretation, to understand what makes single beam (i.e. individual) and sequential irradiation scenarios different from a simultaneous irradiation scenario. Similarly, the study in Ref.^9^ dealt with the effect of coupled electronic and nuclear energy deposition on strain and stress levels in UO_2_ *polycrystals*. It is thus quite apparent that (1) the question (regarding the influence of the grain size on the radiation damage tolerance against simultaneous S_n_ and S_e_) that we are trying to address here is fundamentally very different from those in these studies^1,2,9,24^, and hence (2) such a problem has not at all been investigated earlier.

The investigation of this fundamentally intriguing and technologically relevant question constitutes the work reported in this paper. Cubic zirconia (10 mol% yttria stabilized zirconia) is chosen in the current work because of its importance in the nuclear industry^23,26–28^ and because a good amount of research has already been done with individual low energy and high energy irradiations, thus making available vital results for comparison. Note, *again*, that in the context of nuclear materials, simultaneous irradiations with high energy and low energy particles (and *not* independent and/or sequential low or high energy irradiations (as has been usually done in studies till now)) correspond to the in-service conditions of actual relevance.

## Results

### Pristine samples

**Figure 1:**
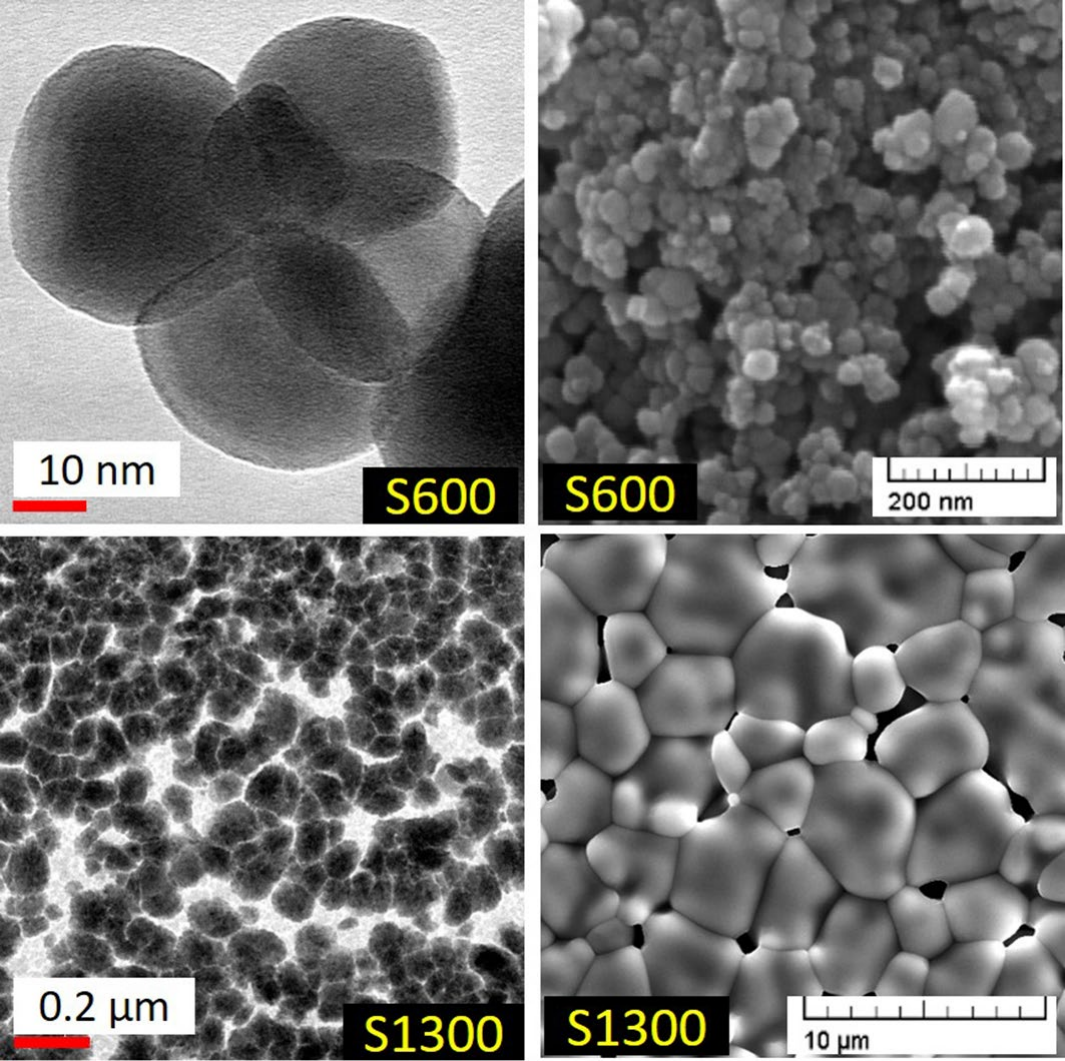
TEM (left column) and SEM (right column) images of pristine S600 and S1300. Adapted from Ref.^21^.

**Figure 2:**
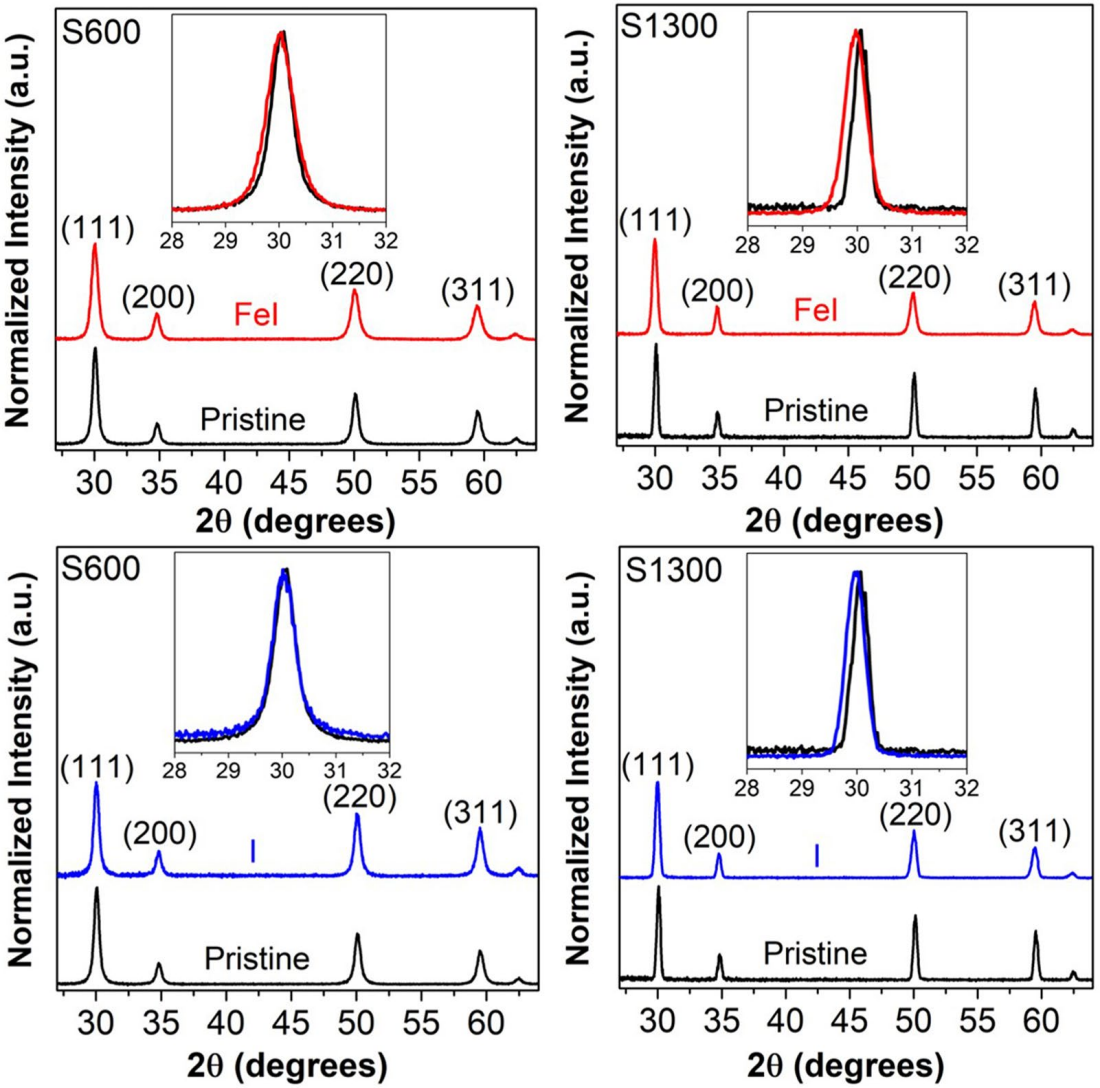
GIXRD patterns of pristine and irradiated S600, S1300. The top row corresponds to the *simultaneous* irradiations (FeI), bottom row corresponds to the *single beam* (i.e., only I) irradiations. Magnified view of the (111) peak for all samples is shown as inset.

The average crystallite/grain size of pristine S600 and S1300 was determined to be ~ 26 nm and ~ 80 nm respectively from TEM imaging (Figure 1) and XRD peak broadening (Figure 2, also see Ref.^21^ for details). SEM imaging (Figure 1) of the pristine samples revealed S1300 to be highly dense and having well-defined particles of size ~ 4.5 ± 2.4 μm, while S600 was found to be less dense and having a much smaller particle size of ~ 38 ± 9 nm. The micron sized S1300 sample can thus be considered as bulk, while S600 is nanosized. The phase of both S600 and S1300 was verified to be the cubic phase from XRD and Raman spectroscopy (Supplementary Information).

### Irradiated samples

The evolution of the glancing incidence XRD (GIXRD) patterns of the S600 and S1300 samples irradiated simultaneously with the 27 MeV Fe and 900 keV I ions are shown in Figure 2. The evolution of the GIXRD patterns upon only 900 keV I irradiation is also shown here. The XRD patterns reveal that, irrespective of the crystallite size and/or type of irradiation (single beam or simultaneous), the XRD peak broadening has increased upon irradiation. This indicates that the irradiations have resulted in the degradation of the long-range crystallinity and periodic structure (i.e., damage). Now in order to evaluate the influence of the grain size on the irradiation induced damage (or conversely the radiation tolerance) against simultaneous S_n_ and S_e_, it is first necessary to have a quantitative estimate of the radiation damage in the various cases. Since the XRD peak broadening is a measure of the (degradation in) crystallinity, the irradiation induced damage is quantified by the relative change in the FWHM upon irradiation. The irradiation damage is accordingly estimated using the equation

1 $$ {\text{damage}} = \frac{{FWHM_{{\left( {111} \right)\_{\text{irradiated}}}} - FWHM_{{\left( {111} \right)\_{\text{pristine}}}} }}{{FWHM_{{\left( {{111}} \right){\text{\_pristine}}}} }} $$

where *FWHM*_*(111)_pristine*_ & *FWHM*_*(111)_irradiated*_ are the FWHM of the (111) diffraction peak for the pristine and irradiated samples Th FWHM of the (111) peak for the pristine and irradiated S600 and S1300 samples, and the corresponding damage are summarized in Table 1. From these values, it is, firstly, apparent that the NC S600 sample is significantly less damaged under the 900 keV I (S_n_) irradiations when compared with the bulk-like S1300 sample. This is in excellent agreement with previous literature (see e.g. Refs.^10,15^) on the effect of grain size on the radiation damage against low energy (S_n_) irradiations. Secondly, irrespective of the crystallite size, the damage after the simultaneous irradiation is greater than the damage after the single beam (i.e., 900 keV I) irradiation. This indicates that no cooperative S_e_/S_n_ effects, that induces a healing of the S_n_ induced damage (like in SiC^1,24^ and MgO^1^), are observed during the simultaneous irradiations in these cubic zirconia samples. This result is again in good agreement with previous literature^1^. Apart from these somewhat expected findings, the important observation is that the NC S600 sample is significantly less damaged under the simultaneous 900 keV I (S_n_) and 27 MeV Fe (S_e_) irradiations. In other words, the XRD results indicate that the NC sample is more radiation damage tolerant than its bulk (-like) counterpart against the simultaneous S_n_ and S_e_ irradiations. This result will be addressed later, in detail, in connection with the general nature of simultaneous irradiations coupled with the role of grain size on the radiation damage tolerance against individual S_n_ and S_e_. Similar observations have been made from Raman spectroscopy measurements as well (results summarized in Table 1, details given as Supplementary Information).

**Table 1:** FWHM of (111) XRD peak, FWHM of F_2g_ Raman band and the damage as calculated from XRD and Raman spectroscopy for all samples. FeI denotes the *simultaneous* irradiations, I denotes the *single beam* (i.e., only I) irradiations.

	FWHM_(111)_ (degree)	Damage (XRD)	FWHM_F2g_(cm^−1^)	Damage (Raman)
S600 pristine	0.52 ± 0.02		96 ± 1	
S600 I	0.56 ± 0.02	7.7%	100 ± 1	4.2%
S600 FeI	0.63 ± 0.02	21.1%	106 ± 1	10.4%
S1300 pristine	0.31 ± 0.02		80 ± 1	
S1300 I	0.39 ± 0.02	26%	89 ± 1	11.3%
S1300 FeI	0.46 ± 0.02	48.4%	98 ± 1	22.5%

**Figure 3:**
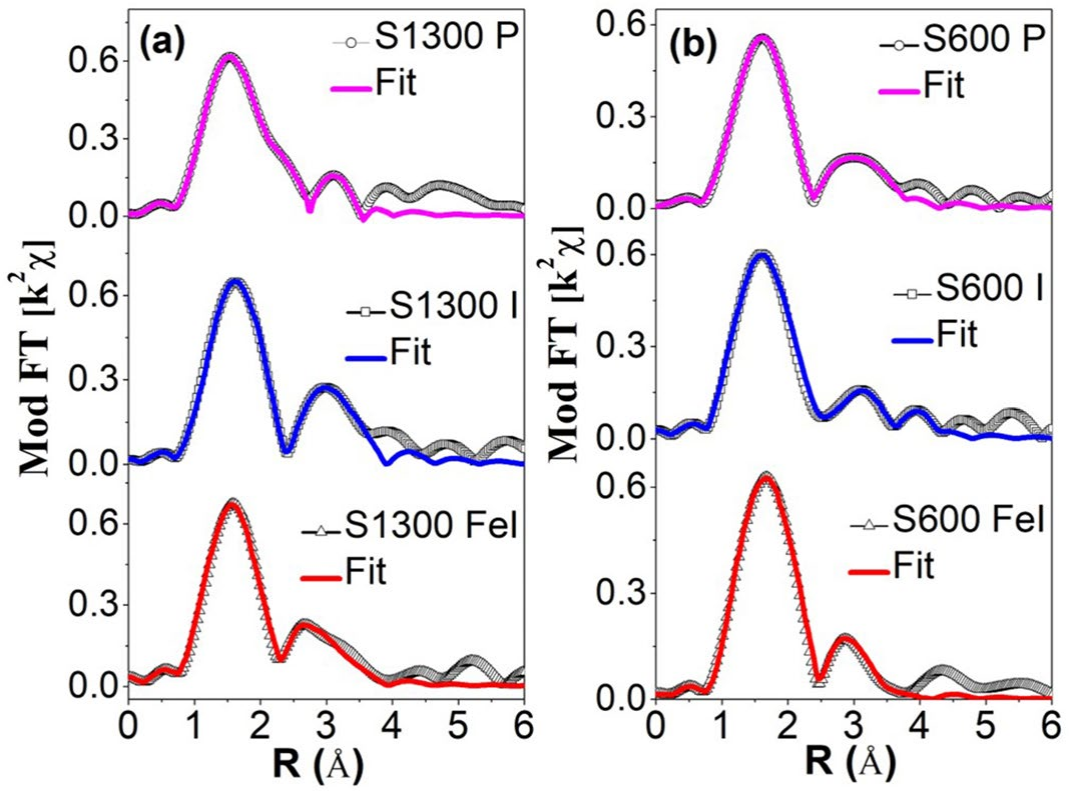
Magnitude of the Fourier transform (FT) of EXAFS functions (k^2^χ*(k)*) for pristine and irradiated S600 and S1300. S1300 P and S600P are the respective pristine samples.

EXAFS was used to obtain information about the changes in the local structure, around a Zr atom, upon irradiation. The magnitude of the Fourier transform (FT) of the *k*^2^ weighted normalized EXAFS functions (i.e., *k*^2^*χ(k)*), for the pristine and irradiated S600 and S1300 samples, is shown in Fig. 3. The *k*-range of 3–7.5 Å^-1^ was used for the FT. Before the FT, the EXAFS function *χ(k)* is obtained from the energy dependent absorption function *χ(E)* using the relation *k* = $\sqrt{\frac{2m(E-{E}_{0})}{{\hslash }^{2}}}$ , where *m* is the mass of electron, *E*_0_ is the absorption edge energy and ℏ is the Planck’s constant. Figure 3 also shows the best fit of the magnitude of the FT; the fitting was performed using cubic zirconia structure in the *R*-space range of 1–3.6 Å corresponding to the first and second nearest neighbour coordination shells. The local structural parameters, i.e. coordination numbers (CN), bond distances (R) and Debye-Waller (DW) factor (σ^2^, indicative of the local disorder), as determined by these fittings are listed in Table 2. In line with previous literature^29,30^, the CN of the pristine samples (both S600 & S1300) was held fixed at 8 and 12 respectively for the first and second coordination shells (note that ideally in cubic zirconia, zirconium has 8 nearest neighbouring oxygen atoms followed by 12 zirconium next nearest neighbour atoms^31^). On the other hand, the CN were floating in the fitting of the irradiated samples since the irradiations can result in the displacement of atoms from their regular sites. Bond distances and DW factors were kept floating in the fitting procedure for all samples.

**Table 2:** Local structural parameters obtained from fitting of the experimental EXAFS data. The uncertainty in the last digit is indicated by the numbers in parentheses. FeI denotes the *simultaneous* irradiations, I denotes the *single beam* irradiations.

	CN_Zr-O_	R_Zr-O_ (Å)	σ^2^_Zr-O_ (Å^2^)	CN_Zr-Zr_	R_Zr-Zr_ (Å)	σ^2^_Zr-Zr_ (Å^2^)
S600 P	8	2.155 (4)	0.0156 (4)	12	3.511 (4)	0.0167 (5)
S600 I	7.3 (3)	2.221 (3)	0.0202 (4)	10.4 (4)	3.543 (5)	0.0218 (5)
S600 FeI	6.3 (4)	2.246 (4)	0.0213 (5)	8.5 (5)	3.573 (5)	0.0226 (6)
S1300 P	8	2.134 (3)	0.0118 (3)	12	3.486 (5)	0.0127 (4)
S1300 I	6.4 (4)	2.154 (4)	0.0157 (4)	10.7 (5)	3.454 (4)	0.0171 (6)
S1300 FeI	5.5 (3)	2.165 (4)	0.0179 (3)	3.4 (5)	3.459 (6)	0.0194 (5)

Upon irradiation, changes in the short-range order are evident for both S600 and S1300. These changes include a decreased CN (Zr-O and Zr-Zr) and an increased DW factor (Zr-O and Zr- Zr). Such irradiation induced changes in the local structure, i.e. decrease in CN and increase in DW factor, are well in agreement with previous literature^29,32,33^. The decrease in the CN indicates vacancy-like (i.e., point) defects, where some of the O and Zr atoms are displaced from their regular sites by the irradiations. The increase in the DW factor upon irradiation implies an irradiation induced increase in the local disorder. Therefore, the EXAFS measurements indicate the creation of local structural damage (creation of vacancy-like defects and/or increase in the disorder) by the irradiations. A thorough observation of the CNs and DW factors reveal that the trend in the irradiation induced damage in the short-range (atomic scale) is the same as the trend of the irradiation damage in the long range (as indicated by GIXRD).

## Discussion

The lower radiation damage in the NC S600 sample as compared to the bulk-like S1300 sample under the 900 keV I (S_n_) irradiations is as per expectations. Since grain boundaries (GBs) are defect sinks, the defects that are produced in the collision cascades upon the S_n_ irradiations are trapped by them (directly (and primarily) in the collision cascades and as well as because of thermal migration from nearby regions to the GBs^15,28^) which results in their removal/reduction. This process of defect removal/reduction by the GBs is much more efficient in S600 as compared to S1300 because: (1) the volume fraction of GBs is higher in the S600 sample, and (2) the possibility of the irradiation induced collision cascades occurring ‘near’ the GBs is also higher (because of its smaller grain size) and hence the defects can more easily interact with the GBs. Moreover, the probability of defects that are not captured directly in the collision cascades reaching the GBs after migrating from the nearby regions is higher too for S600. As such, the S_n_ induced defect concentration is lower in the S600 sample, as compared to S1300, and hence the radiation damage is lesser. In a detailed TEM study on the effect of low energy 400 keV Kr (S_n_ dominant) irradiations on different grain-sized samples of YSZ, the authors had experimentally shown that the defects concentration, after irradiation, is significantly lower in the NC samples as compared to the bulk sample^15^. We can expect similar behaviour in the present case given that the 900 keV I irradiations are in the low energy (S_n_ dominant) regime as well.

Now, in order to interpret the simultaneous irradiation results, it is first necessary to understand the general nature of simultaneous irradiations itself. A simultaneous irradiation scenario is, strictly speaking, simultaneous only when the second ion/particle impact occurs at the same place and in a time period corresponding to the lifetime of the damage event from the previous (i.e. first) ion/particle impact, such that the energy deposition by the second ion perturbs the damage event from the first ion. In other words, a simultaneous irradiation scenario is truly simultaneous only when there is both spatial and time overlap of defect formation/evolution between the two ion beams. It has however been reported that there is essentially no such space-time overlap during simultaneous irradiation^1,2,24^-the probability of such an overlap is actually only about ~ 10^−11^ (see e.g. Ref.^24^). Hence, under simultaneous irradiations, the ‘effect’ of the second ion would not perturb the ‘effect’ of the first ion (during its life-time) and would instead only, in-situ and step-by-step, *follow* it. Therefore, in this structure, simultaneous irradiation can be essentially considered to be equivalent to a series of random sequential irradiations, where the cumulative fluence of these sequential irradiations is equal to the fluence as in the simultaneous irradiation. In-fact, it has been experimentally shown very recently that the simultaneous irradiation scenario is indeed equivalent to multiple small sequential irradiation scenarios^2^. We have therefore interpreted our results within this well-established framework. Accordingly, the simultaneous irradiation (I & Fe) in the present case is considered as a series of irradiations with the I and Fe ions as follows

$$ I \, \left( {\Phi^{I} } \right) \, \& \, Fe \, \left( {\Phi^{Fe} } \right) \, \sim \, I \, \left( {\Phi^{I}_{1} } \right) \, + \, Fe \, \left( {\Phi^{Fe}_{1} } \right) \, + \, I \, \left( {\Phi^{I}_{2} } \right) \, + \, Fe \, \left( {\Phi^{Fe}_{2} } \right) \, + \, \ldots \, + \, I \, \left( {\Phi^{I}_{n} } \right) \, + \, Fe \, \left( {\Phi^{Fe}_{n} } \right) $$

where, Φ^I^ and Φ^Fe^ are the fluence of the 900 keV I and 27 MeV Fe ion beams in the siumultaneous irradiations (Φ^I^ = Φ^Fe^= 10^15^ ions/cm^2^); Φ^I^_j_ and Φ^Fe^_j_ (j = 1 to n) are the incremental fluences of I and Fe respectively that sequentially make up the simultaneous irradiation, and therefore Σ Φ^I^_j_ = Φ^I^ and Σ Φ^Fe^_j_ = Φ^Fe^.

Now, in the case of the simultaneous irradiations, the NC S600 sample will be less damaged as compared to the bulk-like S1300 sample against the 900 keV I ions (fluence Φ^I^_1_) because of its larger fraction of GBs. This is familiar from literature. The GIXRD, Raman spectroscopy & EXAFS results also show that the S600 sample is significantly less damaged than S1300 safter the individual 900 keV I irradiations (although these results are at the total fluence of 10^15^ ions/cm^2^, the trend is expected to be the same after Φ^I^_1_). Upon the subsequent arrival of the 27 MeV Fe ions (fluence Φ^Fe^_1_), the S600 sample is expected to be more damaged than the S1300 sample because of its smaller grain size that results in a more intense thermal spike (see Ref.^21^ for details). The situation is however not as straightforward. Defects in the lattice system can scatter electrons and phonons, thereby resulting in the decrease of lattice thermal conductivity (K_a_) and increase in the electron-phonon coupling strength (g)^34,35^. This in turn would result in a stronger thermal spike in such a defected system as compared to a defect-free (or less defected) system. As shown by Weber et al.^36^, Liu et al.^34^ and Xue et al.^35^, the (pre-)existence of defects in a crystalline system can greatly enhance its sensitivity to electronic energy loss and thereby result in a significantly higher damaged state, upon S_e_ irradiation, as compared to a defect-free system. In the present case, S1300 is significantly more damaged (i.e., more defects) than S600 by the I ions (fluence Φ^I^_1_). Therefore, the intensity of the thermal spike in S1300 after the subsequent arrival of the Fe ions (fluence Φ^Fe^_1_) may be comparable to, or may even be stronger than, that for S600. In other words, although the crystallite size of S600 is much smaller than S1300, the thermal spike generated in it upon Fe irradiation (fluence Φ^Fe^_1_) maybe comparable to that in S1300 because of the existence of significantly more defects in the S1300 system that were created earlier by Φ^I^_1_. Note that the pre-existing defects not only influence the thermal spike but also play a crucial role in the final damage evolution^36,37^. As such, the S1300 sample is affected by both the I (S_n_) and Fe (S_e_) ions, whereas the S600 sample is essentially affected only by the Fe (S_e_) ions. In other words, the damage in S600 is effectively due to S_e_ alone, whereas the damage in S1300 is a superimposition of the damage due to S_n_ and damage due to S_e_ (which itself is a consequence of the pre-existing S_n_ damage). Therefore, the damage in S1300 is higher than in S600 after the I (fluence Φ^I^_1_) + Fe (fluence Φ^Fe^_1_) impact. This process is repeated with the subsequent series of I and Fe ions (fluence Φ^I^_1_ + fluence Φ^Fe^_j_, j = 2 to n). The net result is that S1300 is more damaged than S600 (as evident in GIXRD, Raman spectroscopy & EXAFS).

Thus, the higher radiation damage in the bulk-like/micron-sized sample is ultimately due to its larger grain size (lower density of GBs) that resulted in greater damage against S_n_, and which in turn resulted in significant damage against S_e_ as well. Conversely, the better radiation tolerance of the NC sample is a consequence of its very nature, viz. nano-crystallinity, itself (that results in damage against only S_e_).

**Figure 4:**
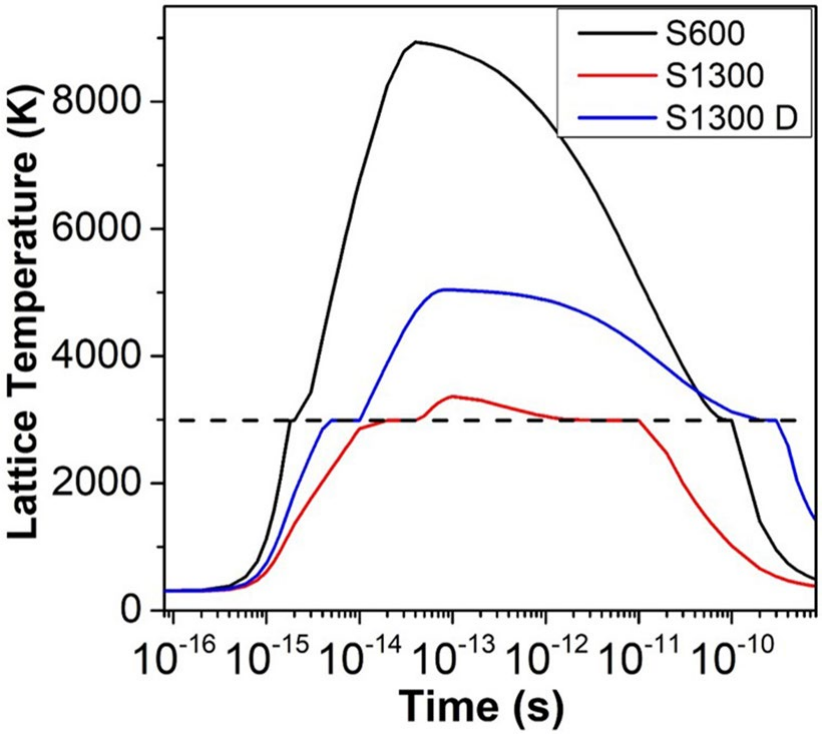
Variation in lattice temperature with time at the center of the ion track for pristine S600, pristine S1300 and S_n_ defected S1300 (S1300 D) upon 27 MeV Fe impact. The dashed horizontal line shows the melting temperature (2988 K).

Thermal spike simulations have been performed to estimate the evolution of the lattice temperature upon the 27 MeV Fe impact. For the sake of completeness, a brief description of the defect/damage production mechanism by the thermal spike process is given as Supplementary Information. Three sets of simulations are performed—(1) S_n_ defected S1300, (2) pristine S1300 (for comparison), and (3) pristine S600. Since S600 is significantly less damaged than S1300 by the 900 keV I ions, we assume that the S_n_ damage in S600 is negligible. Hence the simulations have been performed for pristine S600 only. The effect of grain size (and/or grain boundaries) is introduced into the simulations by considering the effect of grain size on the lattice thermal conductivity and electron-phonon coupling factor^21^. Therefore, for the pristine S1300 and S600 samples, the values of *K*_*a*_ and *g* are taken by considering their respective grain sizes. The values of all the other relevant parameters are the same as taken in Refs.^21,38^. Details of the thermal spike simulations, including the mathematical formulation, is also given as Supplementary Information. To account for the increased scattering of the electrons and phonons from the defects in the S_n_ defected S1300 system, the lattice thermal conductivity of this system is assumed to be an order of magnitude smaller than the pristine S1300 system, and the electron-phonon coupling constant for the system assumed to be 50% larger than the corresponding value for the pristine system^34,36,37,39^. The simulation results are shown in Figure 4, and the values of the maximum thermal spike temperature and thermal spike duration are listed in Table 3. As expected, the thermal spike in the case of the S_n_ defected S1300 sample is notably more intense than in pristine S1300. Comparing S600 and the S_n_ defected S1300 sample, although the maximum thermal spike temperature is higher for S600, the thermal spike duration is significantly (~ 200 ps) shorter. Since the S_e_ induced radiation damage depends on both the (maximum) thermal spike temperature and its duration^21^, the fact that the thermal spike duration is significantly longer for the already S_n_ defected S1300 sample cannot be ignored in analysing the response of the two systems to the energy deposition by the Fe ions. The thermal spike simulations thus suggest that, in addition to the S600 sample, the effect of S_e_ is significant in case of the S1300 sample too under the simultaneous irradiation. This is in agreement with our model of the radiation damage as described above. Combined with the fact that S1300 is significantly damaged by S_n_ as well (as opposed to S600), the simultaneous irradiations result in greater damage in S1300.

**Table 3:** Approximate values of maximum transient lattice temperature and duration of thermal spike at the center of the ion track.

	Maximum temperature	Duration of thermal spike
S600	~ 8900 K	~ 100 ps
S1300	~ 3350 K	~ 10 ps
S1300 D	~ 5000 K	~ 300 ps

## Summary & conclusions

In conclusion, we have elucidated the effect of grain size on the radiation response against ‘simultaneous’ nuclear and electronic energy deposition. The effects of irradiation on the long-range and short-range structure, as the function of grain size, have been investigated with XRD, Raman Spectroscopy and EXFAS. The irradiation induced damage is found to be lower in the NC state when compared with the bulk state. The experimental findings are supported by theoretical thermal spike simulations.

The damage mechanism can be summarized as follows: (1) since there is essentially no spatial and time overlap between the damage events of the two ion beams, the simultaneous irradiation is actually a series of small sequential irradiations with incremental fluences; (2) the NC S600 sample is essentially undamaged in comparison to the bulk-like S1300 against the incremental I ions (because of its smaller grain size); (3) the subsequent impact by Fe ions (with incremental fluence) results in the formation of a damaged state for both S600 and S1300. The S_e_ induced damage in the S600 sample is because of its small grain size. On the other hand, S1300 is also efficiently damaged by the S_e_ (in-spite of its larger grain size) due to the existence of the defects, created earlier by S_n_, that enhances the thermal spike; (4) the irradiation damage in the S1300 sample thus consists of a superposition of the damage by S_n_ and the damage by S_e_ (which itself is a consequence of the pre-existing S_n_ damage), while the S600 sample is essentially damaged only by S_e_. The net result is that S1300 is more damaged than S600. The better radiation tolerance of the NC sample is therefore a consequence of its very nature, viz. nano-crystallinity, itself (that results in damage against S_e_ only).

Finally, it is worth emphasizing that the present study provides the first steps towards the fundamental understanding of the interplay of grain size/GBs and combined S_n_, S_e_ in determining the radiation response (against ‘simultaneous’ S_n_ and S_e_). The fundamental concepts developed here are not merely confined to simultaneous S_n_ and S_e_ deposition, they can also be useful in understanding the material behaviour under other simultaneous (S_n_ and S_n_, S_e_ and S_e_) irradiations. Note that in real-world applications (nuclear reactors (fission and fusion), space vehicles etc.), materials are subjected to multiple particles simultaneously and not independently^2^. In this context, the present results also provide the first realistic evidence in-favour of the potential application of nano-crystalline materials in the nuclear industry where its nano-crystalline nature may result in the strong reduction of the damage production, thus allowing the conservation/prolongation of the physical integrity of the materials subjected to intense (simultaneous S_n_ & S_e_) irradiations. The influence of a couple of vital parameters, viz. irradiation temperature^21,26,38^ and S_e_ to S_n_ ratio, however need to be investigated first for a complete fundamental understanding and before any (potential) applications.

## Methods

### Sample preparation

10 mol% yttria stabilized zirconia (YSZ) powder was prepared by gel combustion method (see Ref.^38^ for details) and then compacted into pellets of diameter ~ 8 mm. The pellets were subsequently heated at 600ºC for 6 hours and 1300ºC for 84 hours with the aim of obtaining different microstructures/crystallite (grain) sizes. The pellets heated at 600ºC and 1300ºC are referred to as S600 and S1300 respectively.

### Ion Irradiation

The S600 and S1300 samples were then *simultaneously* irradiated with 27 MeV Fe ions (S_e_ dominant) and 900 keV I ions (S_n_ dominant) at the JANNUS-Saclay facility^40,41^. The fluence of both the ion species was 10^15^ ions/cm^2^; the irradiations were performed at room temperature with the ion fluxes limited to ~ 10^11^ ions/cm^2^/sec. The S600 and S1300 samples were also irradiated with *only* the 900 keV I ions (i.e., single beam irradiation) keeping all conditions same as in the simultaneous irradiations. The electronic energy loss (S_e_), nuclear energy loss (S_n_) and the projected range of the 27 MeV Fe ions are estimated to be ~ 12 kev/nm, ~ 0.07 keV/nm and ~ 4.3 ± 0.4 µm respectively by SRIM^42^ simulation code; while the corresponding values for the 900 keV I ions are ~ 1.2 keV/nm, ~ 3.1 keV/nm and ~ 185 ± 55 nm respectively. Therefore, during the simultaneous irradiations, only the region up-to a depth of ~ 240 nm is affected by both 27 MeV Fe (S_e_) and 900 keV I (S_n_). Here, it is worth mentioning explicitly that since the focus of the manuscript is the investigation of the radiation damage under *simultaneous* S_e_ and S_n_ deposition, the region irradiated by both the Fe and I ions is of interest and relevance.

### Characterization

GIXRD and Raman spectroscopy measurements, of pristine and irradiated pellets, were performed for structural investigations, including the changes/damage induced by the irradiations. The GIXRD patterns were recorded with Cu K_α_ radiation using a Philips X’Pert Pro diffractometer. The incidence angle was fixed at 0.5º; the probed depth is ~ 140 nm at this angle of incidence. The Raman spectra were recorded using a confocal Renishaw InVia Raman microscope with a laser excitation wavelength of 514 nm and spot size of ~ 1 μm^2^ on the sample surface; the probed depth is a few hundred nanometres. The average crystallite/grain size of the samples was determined prior to irradiation by Transmission Electron Microscopy (TEM) and compared to the crystallite sizes derived from the XRD patterns. This was done in order to independently characterize and estimate the crystallite size with two experimental methods. The TEM images were recorded in plane view mode with 200 keV electrons; for the imaging, the S600 and S1300 pellets were carefully scratched and the obtained powder was dispersed using propanol in TEM grids. Pristine samples were also characterized by field emission Scanning Electron Microscopy (SEM) [MIRA, TESCAN], using a 25 keV electron beam, for information on the surface morphology and particle size. Extended X-ray Absorption Fine Structure (EXAFS) was used to probe the local (i.e., short-range) structural order/environment in the samples, i.e. to investigate the structural changes induced upon irradiation at the atomic scale. The EXAFS measurements were performed at the zirconium (Zr) K-edge in fluorescence mode at the Scanning EXAFS beamline (BL-9) at RRCAT, India. The incidence angle was 90^0^, while the detector was set at the smallest possible glancing angle (with respect to the sample surface, less than 5º) to ensure that only the fluorescence photons arising from the near surface region are collected. With this geometry, the incident penetration depth is ~ 25 microns, however, with the exit angle ≤ 5^0^, the information obtained is from a limited depth of only up-to ~ 150–200 nanometres. Therefore, the depth probed by EXAFS is ~ 150—200 nm. The energy range was calibrated using Zr metal foils. The FEFF 6.0 code^43^ has been used to analyse the EXAFS data. The code facilitates background removal and Fourier transform for deriving the χ(*R)* versus *R* spectra from the absorption data (using ATHENA software^44^). This is followed by generation of a theoretical XAFS spectra from an assumed crystallographic structure and lastly the fitting of the experimental data with the theoretical spectra via ARTEMIS software^44^.

## Supplementary Information

Supplementary Information.
