# Supplementary material for: Grain size effect on the radiation damage tolerance of cubic zirconia against simultaneous low and high energy heavy ions: Nano triumphs bulk
Source: Sci Rep. 2021 May 25;11:10886. doi: 10.1038/s41598-021-90214-6 (PMC8149450; doi:10.1038/s41598-021-90214-6)
Supplement: Supplementary file 1 — Supplementary Information. [file 41598_2021_90214_MOESM1_ESM.pdf]

SUPPLEMENTARY INFORMATION

Grain size effect on the radiation damage tolerance of cubic zirconia against simultaneous low and high energy heavy ions: Nano triumphs bulk

Parswajit Kalita, Santanu Ghosh, Gaëlle Gutierrez, Parasmani Rajput, Vinita Grover, Gaël Sattonnay & Devesh K. Avasthi

❖ Raman Spectroscopy

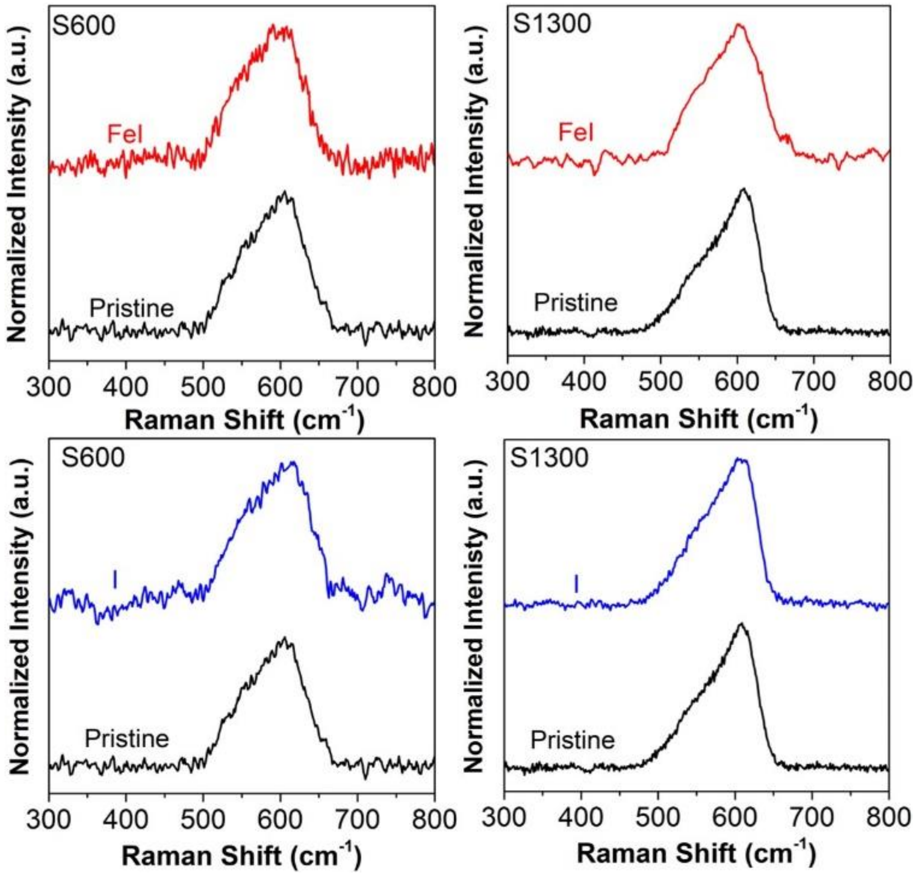

Figure S1: Raman spectra of pristine and irradiated S600 and S1300 showing the F_{2g} band. The top and bottom rows correspond to the *simultaneous* (27 MeV Fe & 900 keV I) and the *single-beam* (only 900 keV I) irradiations respectively.

The Raman spectroscopy measurements of the pristine and irradiated samples were carried out with the aim of investigating the changes brought upon at the microscopic level upon irradiation and to substantiate the GIXRD findings. Figure S1 shows the Raman spectra of unirradiated and irradiated NC S600 and bulk-like S1300 samples. The broad asymmetric band, centered at around 608 cm^{-1}, seen in both the unirradiated samples is assigned to the Raman active F_{2g} mode of Zr-O vibration with O_h^5 symmetry [Refs. 1, 2]. This asymmetry is attributed to the presence of disorder in the YSZ system induced by the doping of Yttrium (Y^{3+}) ions into the zirconia structure [Ref. 2]. The FWHM of the F_{2g} band for pristine and irradiated samples are listed in Table 1 (main article). Amongst other factors, since the width of the Raman band is a measure of the crystallinity, it is apparent that irradiation has resulted in degradation of the crystallinity (peak broadening), i.e. damage, irrespective of the crystallite size and/or type of irradiation. The damage was again quantified using equation (1) [main article] with the FWHM of F_{2g} band of pristine and irradiated samples, and is listed in Table 1 (main article). In agreement with the XRD results, it can be seen that: (i) the NC S600 sample is

significantly less damaged than the bulk-like S1300 sample under the 900 keV I irradiations, (ii) for both S600 and S1300, the damage after the simultaneous 900 keV I and 27 MeV Fe irradiations is higher than that after the individual 900 keV I irradiation, and (iii) crucially, the NC S600 sample is significantly less damaged under the simultaneous 900 keV I and 27 MeV Fe irradiations as compared to the bulk-like S1300 sample. It may be noted that the damage values as estimated from Raman spectroscopy is lower than the corresponding values estimated via GIXRD. This may be due to the reason that the information depth of the two techniques is different – the Raman penetration depth is expected to be more than the ~ 140 nm probed by GIXRD and some part of the detected Raman signal may have originated from the part of the samples untouched by the 900 keV I beam. Nevertheless, the important point is that the trend in the values, estimated via both techniques, is the same.

❖ Thermal Spike process & simulations

Mechanism of defect/damage production by thermal spike:

In case of high energy heavy ion irradiations, the energy loss of the incident ions is dominated by S_e (i.e., $S_e >> S_n$). In this case, the modification (damage) of the target material upon energy transfer from the incident ions to the target atoms can be understood by the 'thermal spike' model.

The thermal spike model considers the target material as a two-component medium: the electrons and the lattice. The incident ion initially transfers its energy to the electrons which results in an increase of the electronic temperature. The energy then gets transferred from the electrons to the lattice via electron-phonon coupling. This results in a sudden and large increase (i.e., 'spike') in the lattice temperature. When the lattice temperature exceeds the melting point of the material, a molten state is formed in a localized cylindrical zone of few nano-meters radius around the ion path which subsequently gets quenched rapidly by thermal conduction. This sudden heating followed by the rapid quenching results in the formation of damaged (defected and/or amorphous) matter within the cylindrical region – known as the ion track. The entire process is governed by two coupled partial differential equations in cylindrical geometry – as a consequence of the energy $B(r, t)$ deposition in the electronic sub-system by the incident ions, the temperature evolution of the electronic and lattice sub-systems is given by

$$C_e(T_e)\frac{\partial T_e}{\partial t} = \frac{1}{r}\frac{\partial}{\partial r}\left[rK_e(T_e)\frac{\partial T_e}{\partial t}\right] - g(T_e - T_a) + B(r,t)$$

$$\rho C_a(T_a)\frac{\partial T_a}{\partial t} = \frac{1}{r}\frac{\partial}{\partial r}\left[rK_a(T_a)\frac{\partial T_a}{\partial t}\right] + g(T_e - T_a)$$

where, T_e and T_a are the electronic and lattice temperatures; ρ is the material density; C_e, C_a, K_e and K_a are the specific heats and thermal conductivities of electronic and lattice subsystems respectively; g is the electron phonon coupling constant; r, t are the radial distance from the ion path and time respectively; and $\iint 2\pi r\, B(r,t)\, drdt = S_e$. The electron-phonon coupling constant governs the efficiency of the energy transfer from the electrons to the lattice and hence determines the temperature spike in the lattice; while the heat/energy dissipation in the lattice, after the temperature spike, is governed by the lattice thermal conductivity. The

thermal spike in a material, and hence the S_e induced damage, is therefore critically governed by its lattice thermal conductivity and electron-phonon coupling strength.

Effect of grain size & mathematical formulation:

Now, the electron-phonon coupling constant is given by the relation [Ref. 3]

$$g = \frac{9 n_e k_B^2 T_D^2 v_F}{16 \lambda T E_F} \quad \text{……….. (s1)}$$

where, n_e is the number density of electrons, k_B is Boltzmann's constant, T_D is the Debye temperature, v_F is the Fermi velocity, E_F is the Fermi energy, T is the environmental temperature and λ is the mean diffusion length of the excited electrons i.e., λ is the electron-phonon mean free path. It is clear from the above equation that λ is the key parameter that determines the strength of the electron-lattice interaction and hence the temperature spike in the lattice. Now, it is well known that grain boundaries (GBs) act as a barrier for electron transport. Hence, the mean diffusion length of the electrons (λ) is strongly influenced by the grain/crystallite size. This is because as the size decreases and/or the amount of GBs increases, the grain boundary scattering increases which consequently results in reduction of the electron-phonon mean free path. The relation between the mean diffusion length λ and the grain/crystallite size (d) is given by [Ref. 3]

$$\lambda = \frac{\alpha d (1-R)}{R} \quad \text{………. (s2)}$$

where α is the angle between the material plane and the velocity vector of the electrons and R is the reflection coefficient of electrons striking the GBs. Thus, due to the smaller grain sizes, λ will be smaller in the nano-crystalline (NC) systems as compared to bulk systems, which will result in stronger g and consequently higher density of deposited energy into the lattice. As a result, the transient lattice temperature will be higher for NC systems as compared to bulk systems.

Also, since GBs act as an obstacle for heat transport by scattering and confining the motion of phonons, the lattice thermal conductivity is strongly dependent on the grain size. In particular, as the size decreases, the density of GBs increases, and the scattering and confinement of the phonons becomes significant. This leads to a decrease in the thermal conductivity. As such, the dissipation of the heat after the ionizing thermal spike will be less effective in NC systems, compared to bulk systems, resulting in longer duration of the thermal spike. The effective thermal conductivity for a material with grain/crystallite of size 'd' can be described by [Ref. 3]

$$K = \frac{\dfrac{K_0}{1+\Lambda_0/d^{0.75}}}{1+\dfrac{R_k}{d}\left[\dfrac{K_0}{1+\dfrac{\Lambda_0}{d^{0.75}}}\right]} \quad \text{………… (s3)}$$

where K_o is the single crystalline thermal conductivity, Λ_o is the single crystal phonon mean free path and R_k is the Kapitza thermal resistance. Λ_o is given by [Ref. 3]

$$\Lambda_0 = \frac{20T_m a}{\gamma^2 T}$$

where T_m is the melting temperature, a is the lattice constant, γ is the Gruneisen constant and T is the environmental temperature. In the present case for YSZ, Λ_o = 25 nm with T = 300K (room temperature irradiations), T_m = 2988 K, a = 0.513 nm and γ = 2.

Therefore, as a result of the combination of these two factors, viz. (i) strong capability of energy transfer from the electronic system to the lattice (enhanced 'g') and (ii) poor dissipation of the energy in the lattice (low thermal conductivity), the thermal spike induces much higher transient lattice temperatures with longer durations in NC systems (e.g., S600) as compared to bulk / bulk-like systems (e.g., S1300). Consequently, significantly larger damage will be produced in NC systems as compared to bulk systems upon high energy heavy ions irradiation. This has also been experimentally observed in our previous work [Ref. 3]. In the present work, for the 27 MeV Fe ions irradiation, the effect will be qualitatively similar - i.e., the S600 sample would experience a more intense thermal spike as compared to S1300 upon Fe ion impact, and would thus be more damaged (note that the 27 MeV Fe ions are high energy heavy ions with $S_e >> S_n$; moreover, S_e is of the same order in both Ref. 3 and the present study). Thermal spike simulations (Figure 4 & Table 3 in the main article) show that the S600 sample indeed experiences much higher transient lattice temperature with longer duration in comparison to S1300 after 27 MeV Fe irradiation. Note that this discussion is valid in case of independent 27 MeV Fe irradiation and not for the simultaneous 900 keV I & 27 MeV Fe irradiations. In case of the simultaneous irradiations, the situation is not as straightforward as described above. As outlined in the main article, during the simultaneous irradiations, the intensity of the thermal spike in S1300 after Fe ion impact may be comparable to, or may even be stronger than, that for S600 because of the existence of defects (in S1300) created earlier by 900 keV I.

Details of the simulations:

It is also clear from the above description that the grain size is a vital factor influencing the thermal spike process and thus the radiation damage upon high energy heavy ions irradiation. Therefore, the grain size of the material must be considered in thermal spike simulations for a correct description / understanding of S_e induced damage. In the present work, the effect of grain size has been introduced in the simulations by considering the effect of grain size on the lattice thermal conductivity and electron-phonon coupling factor. The details are presented below.

Equation (s1) has been used to estimate the electron phonon coupling factor (g). λ for the different samples (S600, S1300) is estimated using equation (s2). As the values of α and R are not known, the following assumptions have been made [Ref. 3]: (i) α and R are constant for all the samples, i.e. constant for all the crystallite sizes, and (ii) the sample with the largest crystallite size behaves like bulk single-crystalline. Therefore, for S1300 (with d = 80 nm), λ= 4.5 nm [Ref. 3]. Using this value of λ for S1300, the value of λ is

calculated to be ~ 1.5 nm for S600 (with d = 26 nm). Equation (s3) was used to calculate the thermal conductivity of S600 and S1300 (the K_o values are taken from [Ref. 4]) with Λ_o = 25 nm. The values of the parameters used in the simulations are tabulated below -

	**S600**	**S1300**
**K_a**	$1.37*10^{-2}$ W/cm K	$2.51*10^{-2}$ W/cm K
**C_a**	0.4572 J/g K	0.4572 J/g K
**C_e**	1 J cm^{-3} K^{-1}	1 J cm^{-3} K^{-1}
**K_e**	2 J cm^{-1} s^{-1} K^{-1}	2 J cm^{-1} s^{-1} K^{-1}
**g**	0.18 J cm^{-3} s^{-1} K^{-1}	0.06 J cm^{-3} s^{-1} K^{-1}
**T (initial)**	300 K	300 K
**S_e**	12 keV/nm	12 keV/nm

It is important to mention here that for the thermal spike simulations, we have used the thermal spike simulation code provided by Dr. M. Toulemonde (the code is essentially a solution to the two coupled partial differential equations that describes the thermal spike process). As such, we did not set any boundary conditions ourselves. In the code/simulations, the limits of integration over space and time of the energy density per unit time supplied by the incident ions (i.e., B) is taken as $t = 0$ to $t = \infty$ and $r = 0$ to $r = r_m$ where r_m is the maximum projected range of electrons perpendicular to the ion path [see Ref. 5 for details].

Lastly, as outlined in the main article, to account for the increased scattering of the electrons and phonons from the defects in the S_n defected S1300 system (S1300D), the lattice thermal conductivity of this system is assumed to be an order of magnitude smaller than the pristine S1300 system (S1300), and the electron-phonon coupling constant for the system assumed to be 50% larger than the corresponding value for S1300.

References:

1) Cai, J., Raptis, C., Raptis, Y. S. & Anastassakis, E. 'Temperature dependence of Raman scattering in stabilized cubic zirconia.' Physical Review B 51, 201 (1995).
2) Kosacki, I., Petrovsky, V., Anderson, H. U. & Colomban, P. 'Raman spectroscopy of nanocrystalline ceria and zirconia thin films.' Journal of the American Ceramic Society 85, 2646-2650 (2002).
3) Kalita, P. et al. 'Investigating the effect of material microstructure and irradiation temperature on the radiation tolerance of yttria stabilized zirconia against high energy heavy ions.' Journal of Applied Physics 125, 115902 (2019).
4) Wilkes, K.E., Dinwiddie, R.B., & Graves, R.S. 'Thermal Conductivity' 23, CRC Press 1996.
5) Wang, Z.G. et al. 'The Se sensitivity of metals under swift-heavy-ion irradiation: a transient thermal process.' J. Phys.: Condens. Matter 6 6733-6750 (1994).
